# Custom Hydroxyapatite‐Coated Stem Collar With Extracortical Plate Provides Excellent Long‐Term Results Against Aseptic Loosening in Revision, Including Short‐Segment, Endoprosthetic Tumour Reconstruction

**DOI:** 10.1002/cnr2.70254

**Published:** 2025-06-25

**Authors:** Patrick Qi Wang, Kwok Chuen Wong

**Affiliations:** ^1^ Division of Orthopaedics, Department of Surgery Université de Montréal Montreal Canada; ^2^ Department of Orthopaedics and Traumatology The Chinese University of Hong Kong Hong Kong SAR

**Keywords:** aseptic loosening, extracortical bone bridge, extracortical plate, hydroxyapatite, revision, short‐segment

## Abstract

**Background:**

Aseptic loosening from endoprosthetic reconstructions following bone tumour resection is a major issue, especially in the revision/multiple revisions settings. The objective was to present long‐term outcomes of revision limb salvage surgery using a custom hydroxyapatite‐coated stem collar with an extracortical plate at the bone‐implant junction specifically designed to prevent aseptic loosening.

**Methods:**

Fifteen (15) patients with an initial extremity bone tumour resection and reconstruction who underwent revision surgery utilizing this implant specification between 2004 and 2016 were included. Multiple prior surgeries and short‐segment stem fixation (< 100 mm) were observed in six and nine patients, respectively. Outcomes of interest were rates of aseptic loosening and radiographic evidence of osseointegration along with clinical and functional outcomes.

**Results:**

At mean 12‐year follow‐up (range 6.5 to 18), no patient had evidence of radiographic or clinical aseptic loosening. The distal femur location (*p* = 0.044), endoprosthetic reconstruction as index procedure (*p* = 0.026), mechanical failure as reason for revision (*p* = 0.0047) and additional fixation to the extracortical plate (*p* = 0.041) were associated with higher bone ingrowth scores. All but two patients, who had mild pain only, were pain‐free. Joint range of motion (*p* < 0.0001) and limb‐length discrepancy (*p* = 0.021) significantly improved. The mean Musculoskeletal Tumor Society score was 26.1 (excellent).

**Conclusions:**

This study demonstrated excellent results in preventing long‐term aseptic loosening with this custom implant specification, which is useful particularly for revisions/multiple revisions and short‐segment fixation. However, further larger scale and multicentric studies are needed to validate the data to the broader population.

**Abbreviations**
AP: anterior–posterior
HA: hydroxyapatite
I&D: irrigation and debridement
LLD: limb‐length discrepancy
MSTS: Musculoskeletal Tumor Society
PJI: prosthetic joint infection
VBG: vascularized bone graft

## 1 Introduction

A pooled analysis including 49 studies and 2721 patients revealed an overall 12% rate of aseptic loosening at the bone‐implant interface following endoprosthetic tumour reconstruction of the extremities with a mean follow‐up of 94 months [1] and this number increases up to 49% in patients who survive beyond 25 years [2]. Although there is a paucity of data regarding re‐revisions of tumour endoprosthesis for aseptic loosening, one study showed a 29% rate for distal femoral replacements [3]. Over the years, various techniques have been described to improve osseointegration, including the use of porous‐ or hydroxyapatite (HA)‐coated stem collars, extracortical bone‐bridging techniques, vascularized bone graft (VBG), larger cemented stem‐to‐bone ratio and press fit cementless stems [4, 5, 6, 7, 8, 9, 10, 11]. From a functional outcomes point of view, preservation of native bone and joint remains ideal. Therefore, various custom prostheses have been developed for short‐segment fixation, including custom cross‐pin fixation, compressive stem osseointegration, uncemented spreading stems and custom extracortical plates [7, 12, 13, 14].

In a setting where vascularity and bone growth environment may be compromised due to multiple surgeries and large surgical beds from previous tumour resections [9], the preference from this institution, when feasible, is to revise the bone‐implant interface using a custom HA‐coated stem collar with an extracortical plate for patients who had an index tumour resection and failed reconstruction procedure. When possible, a vascularized extracortical bone bridge is created at the junction. HA‐coated collars have demonstrated superior osseointegration, whereas non‐coated collars often have evidence of healing with fibrous tissue [5, 15]. Meanwhile, a custom extracortical plate allows better rotational stability and enhances the initial fixation and stability [7, 16, 17]. When feasible, a vascularized extracortical bone bridge using in situ bone still attached to the soft tissues is utilized to provide better blood supply and bone growth environment [9, 18].

Given the burden of multiple surgeries and the increased risk of infection with each surgery, this technique aims to provide a reliable and durable solution to mitigate the risks of subsequent aseptic loosening. Therefore, this study presents the long‐term outcomes of this custom implant specification in revisions (and re‐revisions) of tumour endoprosthetic reconstruction surgery with the primary goal of reducing the rate of aseptic loosening.

## 2 Methods

Consecutive patients between January 2004 and December 2016, who underwent revision or re‐revision surgery utilizing a custom endoprosthesis with a HA‐coated stem collar and extracortical plate at the bone‐implant junction following failed index (initial) extremity primary bone tumour resection and reconstruction were retrospectively reviewed. The minimum follow‐up time was six months. Patient exclusion criteria were 1) index procedure for non‐oncologic reasons (e.g., trauma, degenerative or inflammatory conditions, neuromuscular disease), 2) index procedure for management of primary soft tissue malignancy with bone extension, 3) surgery with the custom endoprosthesis for the treatment of a metastatic bone lesion, 4) non‐extremity locations and 5) patients lost to follow‐up within six months postoperatively.

All surgeries were performed at the Prince of Wales Hospital (Hong Kong), a musculoskeletal oncology referral centre. Approval from the *Joint Chinese University of Hong Kong‐New Territories East Cluster* Clinical Research Ethics Committee was obtained. Informed consent was obtained from all patients or their parents/legal guardian.

**FIGURE 1:**
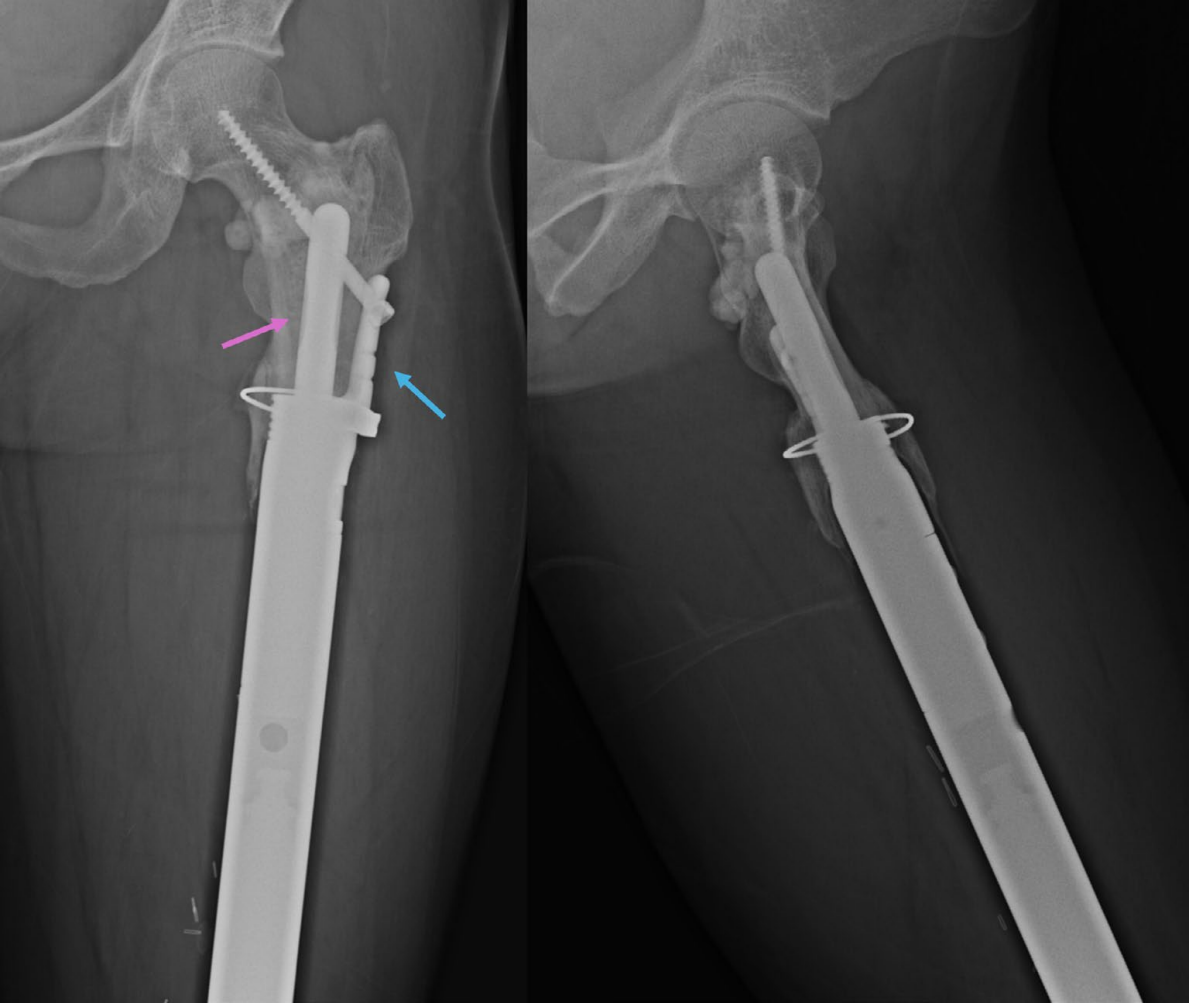
Plain radiographs of a distal femur endoprosthesis with a HA‐coated stem collar, short‐segment cemented stem (purple arrow), extracortical plate (blue arrow) and screw fixation into the femoral head. The stem length is 54 mm, and the plate length is 36 mm.

Baseline characteristics retrieved for each patient include demographics (age at index surgery and final revision, sex), oncologic history (location, index diagnosis, index tumour volume and length, index surgery, index chemotherapy and/or radiation), revision history (time between index procedure and final revision, multiple revisions and reason for revision) and surgical/implant information (standard‐ vs. short‐segment fixation, stem‐to‐bone ratio, plate fixation (cable, screw or none), length of extracortical plate‐to‐length of residual native bone ratio, additional bone resection and bone graft method). Tumour volume was calculated based on the previously described ellipsoidal mass by multiplication of (π/6), craniocaudal, anterior–posterior (AP) and right‐to‐left maximum lengths [19]. *Short‐segment* was defined as a native bone stock that can only accommodate a less than 100 mm stem [14]; see Figure 1. The stem‐to‐bone ratio was measured by dividing the stem width by the cortical diameter of the bone diaphysis or the area closest to the diaphysis in the case of short‐segment stems [4, 20]. The residual native bone length was measured from the osteotomy site to the hip centre, the ankle joint and the elbow joint, for the distal femur (including the intercalary femur), proximal tibia and proximal humerus reconstructions, respectively, based on immediate postoperative plain radiographs. In this study, the term final revision refers to the endoprosthetic revision with the HA‐coated stem collar and extracortical plate. This excludes revisions due to further complications from the final revision surgery, which is analysed separately.

### 2.1 Custom Endoprosthesis Design and Surgical Technique

**FIGURE 2:**
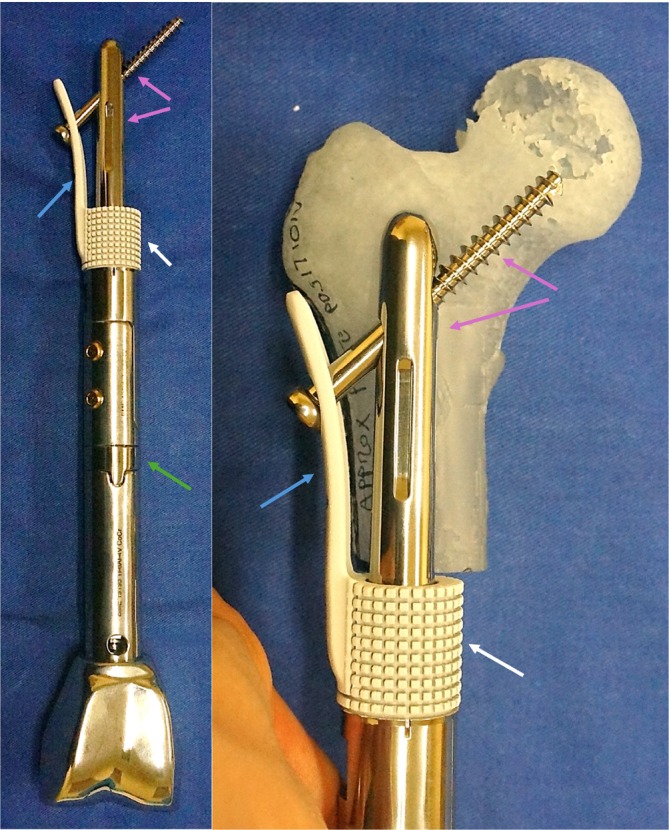
Custom distal femur endoprosthesis with HA‐coated stem collar (white arrows) and an extracortical plate (blue arrows), short‐segment stem with femoral head and neck screw fixation (purple arrows). Intraoperative endoprosthesis length adjustment was performed with different lengths of C‐collars (green arrow) according to the soft tissue and vascular status of the operated limb.

**FIGURE 3:**
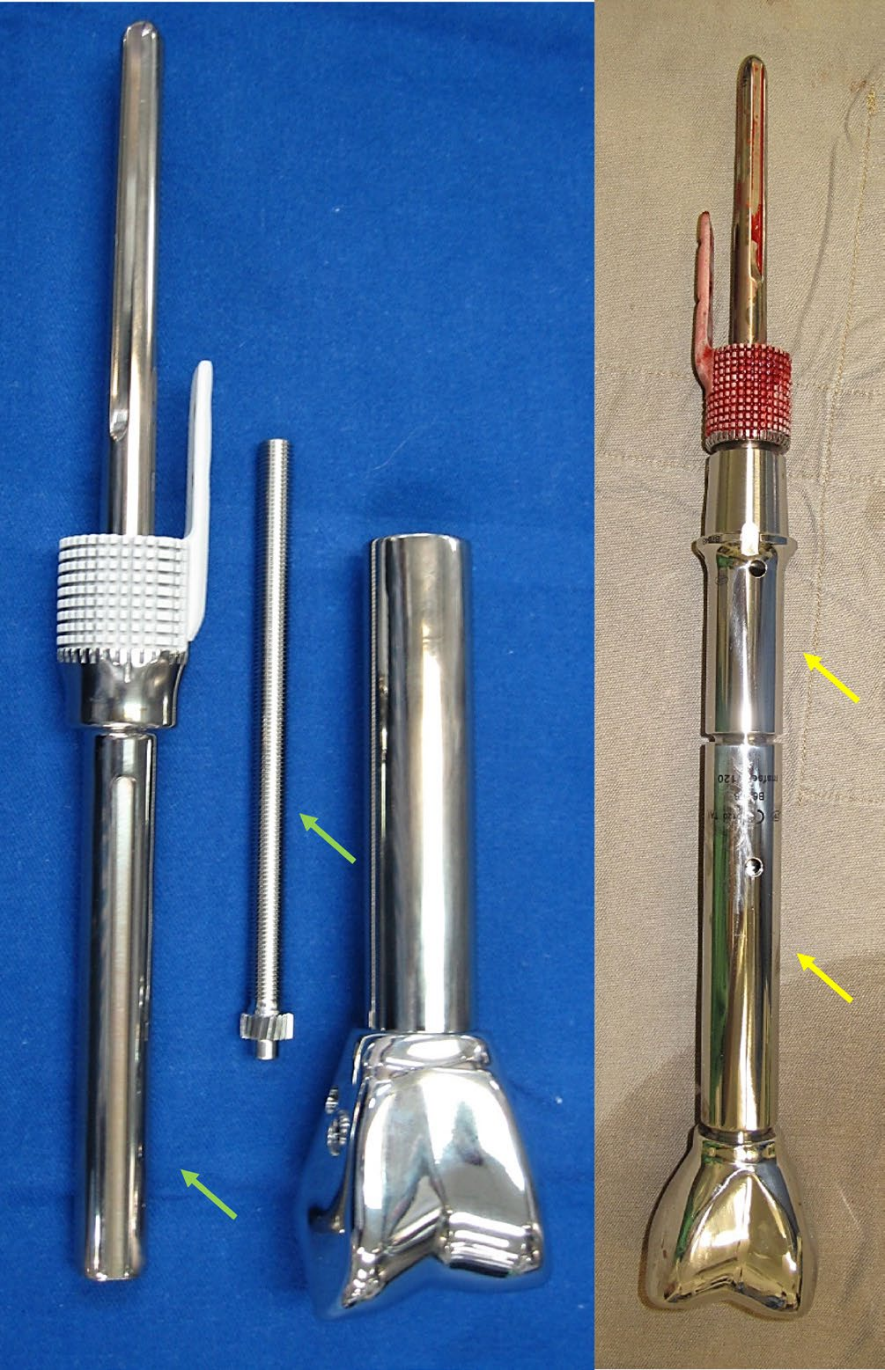
Custom HA‐coated stem and extracortical plate assemblies. Minimally invasive extendable distal femur endoprosthesis (green arrows) or off‐the‐shelf modular distal femur endoprosthesis (yellow arrows) were used for leg length adjustment.

Based on preoperative imaging of the affected extremity, the surgical team determined the bone resection at the level where the cortical bone was normal to accommodate the cemented stem of the custom endoprosthesis or to facilitate the ease of removing the well‐fixed previous implant. As the bone medullary canals were compromised by the failed previous limb salvage reconstructions, extracortical plates were added to the stem collars to improve the primary fixation of the custom endoprostheses with additional rotational stability. The extracortical plates were located on the tension side of the bone‐implant junction. The initial designs accommodated unicortical screw fixation via the plate, while the later designs incorporated grooves for cerclage wiring to reduce bone stress risers or shielding. All stem collars and extracortical plates were also coated with HA, increasing the contact area to the normal bone and enhancing the secondary osseointegration at the bone‐implant junction for better implant longevity. As the patients with failed limb salvage reconstructions had extremity shortening and reduced joint movement, HA‐coated stem collars and extracortical plates were designed to have a modular attachment to the remaining endoprostheses whenever intraoperative adjustment of the leg length and joint motion after soft tissue releases were required; see Figures 2 and 3 for various lengthening techniques. The custom endoprostheses were designed and manufactured by the former Stanmore Implants Worldwide Limited (Elstree, United Kingdom). The surgical team approved the engineers' designs of the custom endoprostheses that met the requested surgical requirements before the actual endoprosthetic manufacturing.

**FIGURE 4:**
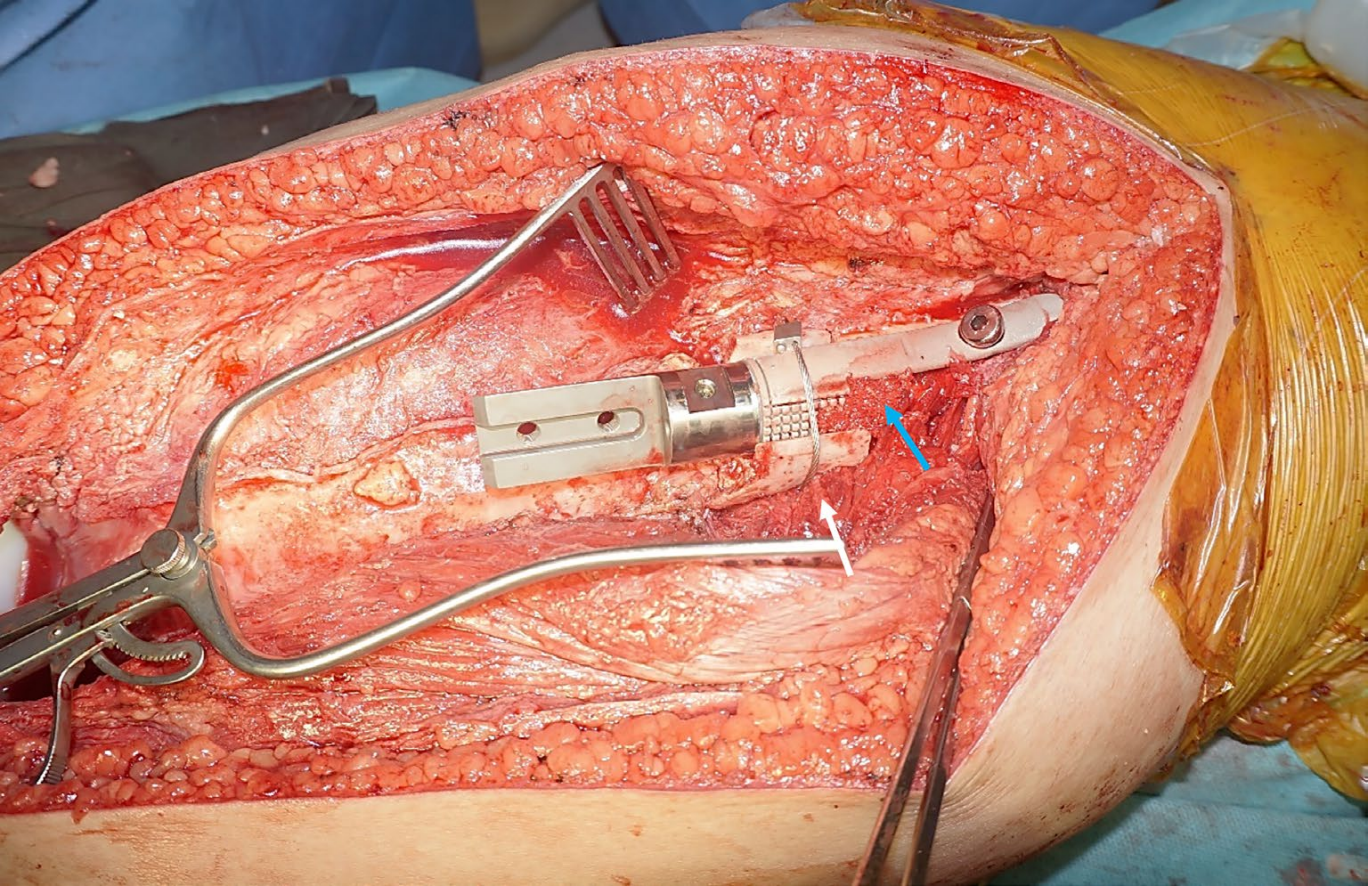
Custom HA‐coated stem collar and extracortical plate with femoral head and neck screw were inserted. A segment of remaining cortical bone with soft tissue attachment was split and secured with cerclage wire (white arrow). Additionally, cancellous bone grafts (blue arrow) obtained while reaming the proximal femoral canal were added to the bone‐implant junction to enhance secondary osseointegration.

Upon confirming that there was no evidence of infection or, if applicable, that the previous infection was completely eradicated, the former implants and/or bone grafts were extracted. In cases where a previous cemented endoprosthesis was utilized, the cement mantle was removed from the medullary bone canal using cement removal instruments. The bone was osteotomized at the planned level during the endoprosthetic design. The medullary canal was further reamed to the desired diameter before the new custom endoprosthesis was implanted with cement. Excess cement was removed to ensure that no cement interfered at the HA‐coated bone‐implant junction. Additional screws or cerclage wires were added to the extracortical plates if necessary to enhance primary fixation and stability. However, additional fixation was not routinely performed, and final decisions were made on a case‐by‐case basis. When possible, any retained bone after the osteotomy with preserved soft tissue attachment was split and then wrapped around the bone‐implant junction for better primary stability and secondary osseointegration; see Figure 4 demonstrating a junctional bone grafting technique. The final leg length was adjusted intraoperatively with the endoprosthetic components according to the final soft tissue and vascular status, as well as the amount of nearby joint motion allowed. Soft tissue flap coverage requirement was determined preoperatively and intraoperatively on an individual basis. Protected or full weight bearing was allowed for each patient, and range of motion was initiated early postoperatively as soon as the wound healed.

### 2.2 Outcomes of Interest

**FIGURE 5:**
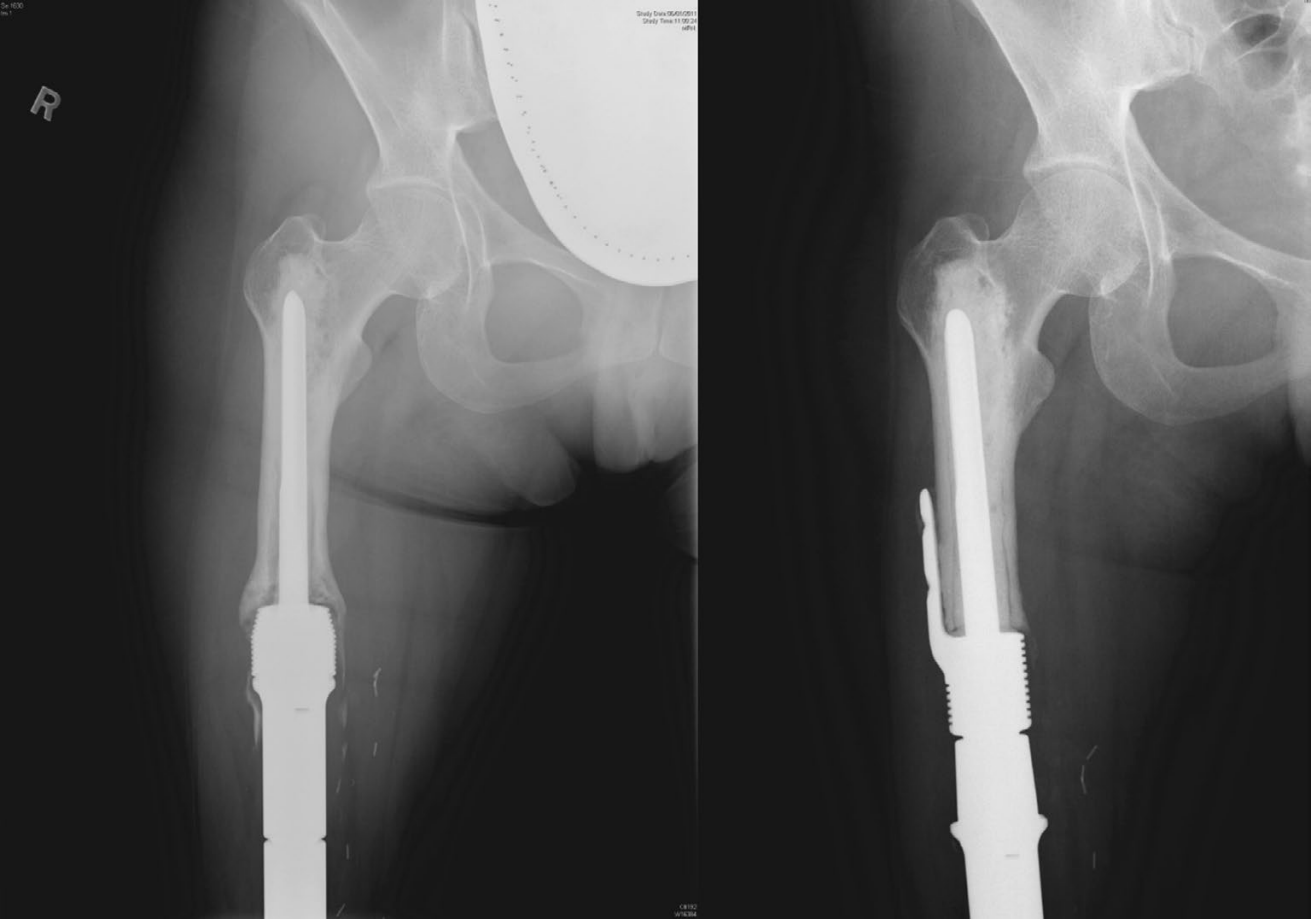
Left: Plain radiograph of aseptic loosening of a previous distal femur endoprosthesis following failed allograft reconstruction for the treatment of osteosarcoma. Infectious workup was negative. Right: Immediate postoperative plain radiograph of the revision to a distal femur endoprosthesis with HA‐coated stem collar and an extracortical plate.

**FIGURE 6:**
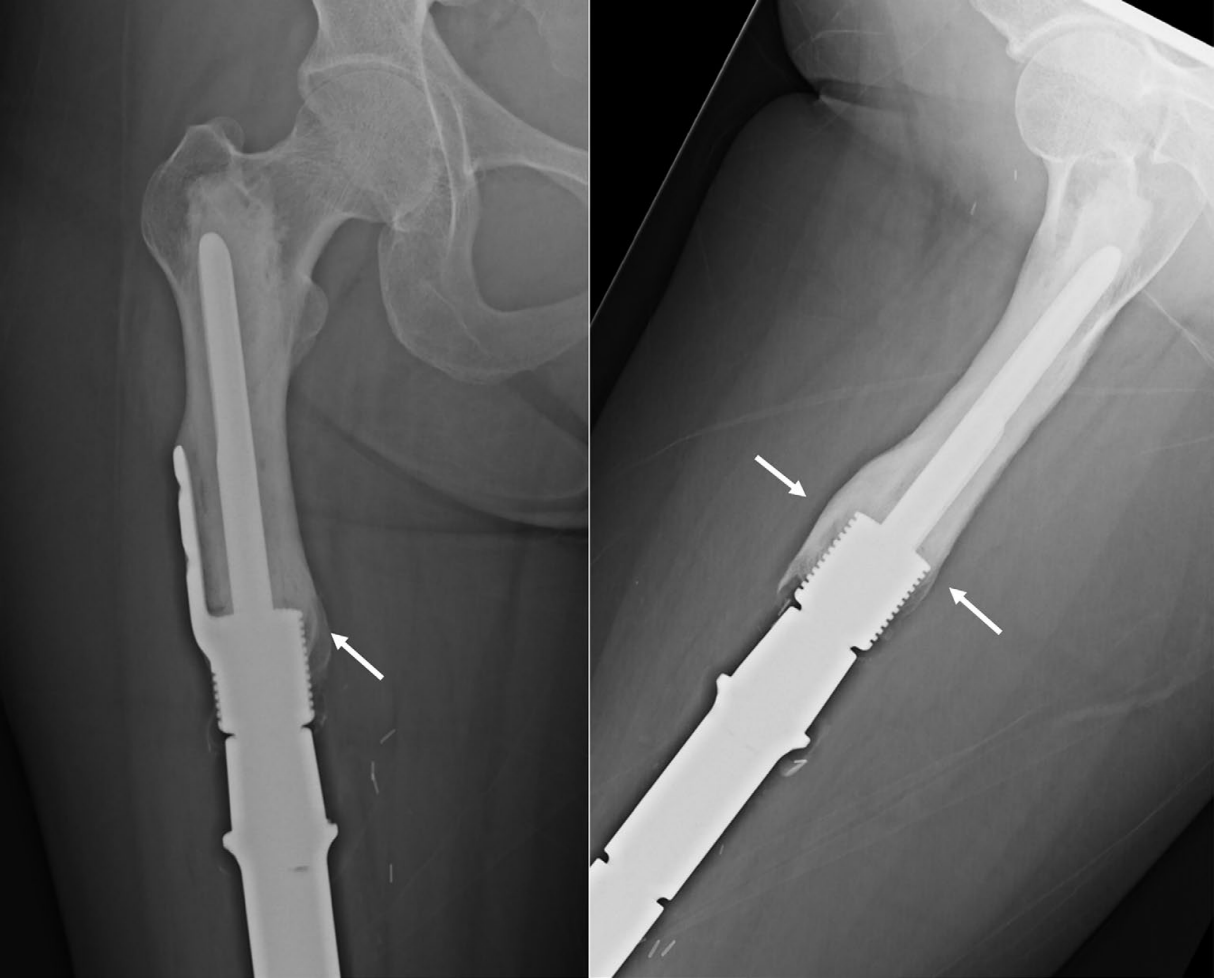
Most recent plain radiographs of patient in Figure 5 showing extracortical bone bridge ingrowth (white arrows).

**FIGURE 7:**
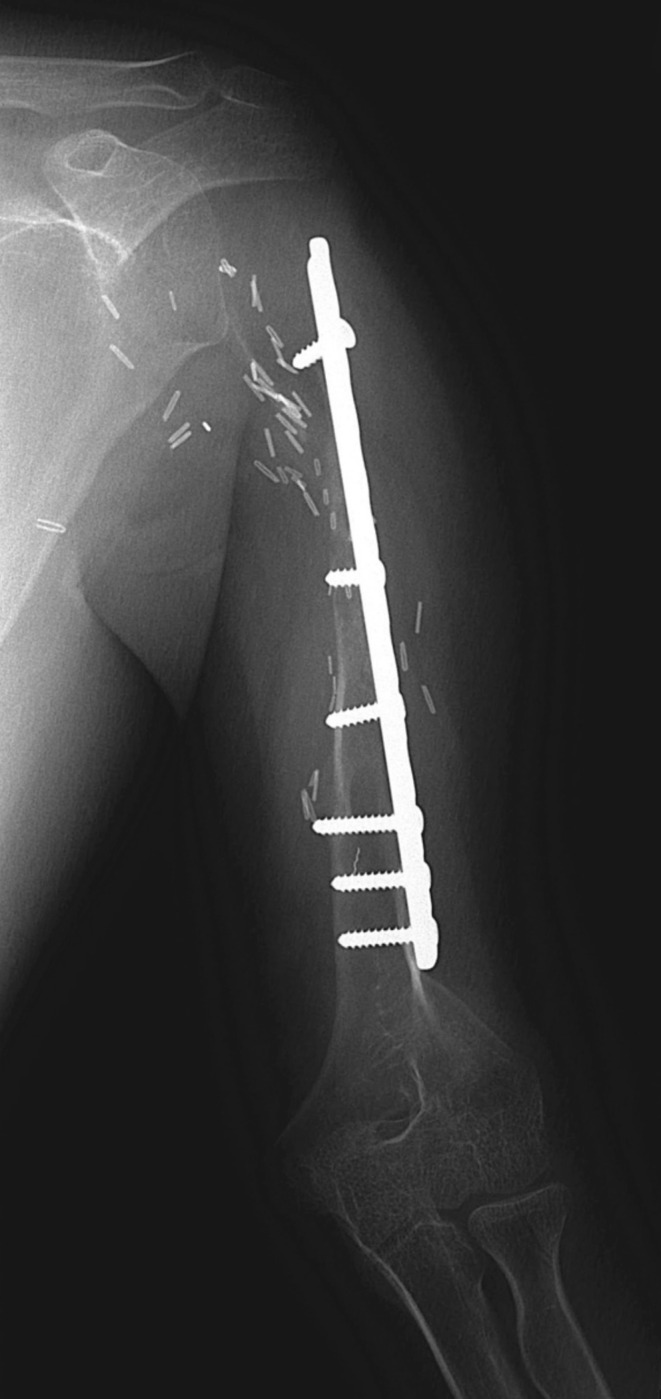
Plain radiograph of a paediatric patient who underwent fibular vascularized bone graft reconstruction following proximal humerus resection for Ewing's sarcoma. This was complicated by traumatic graft fracture and resorption.

**FIGURE 8:**
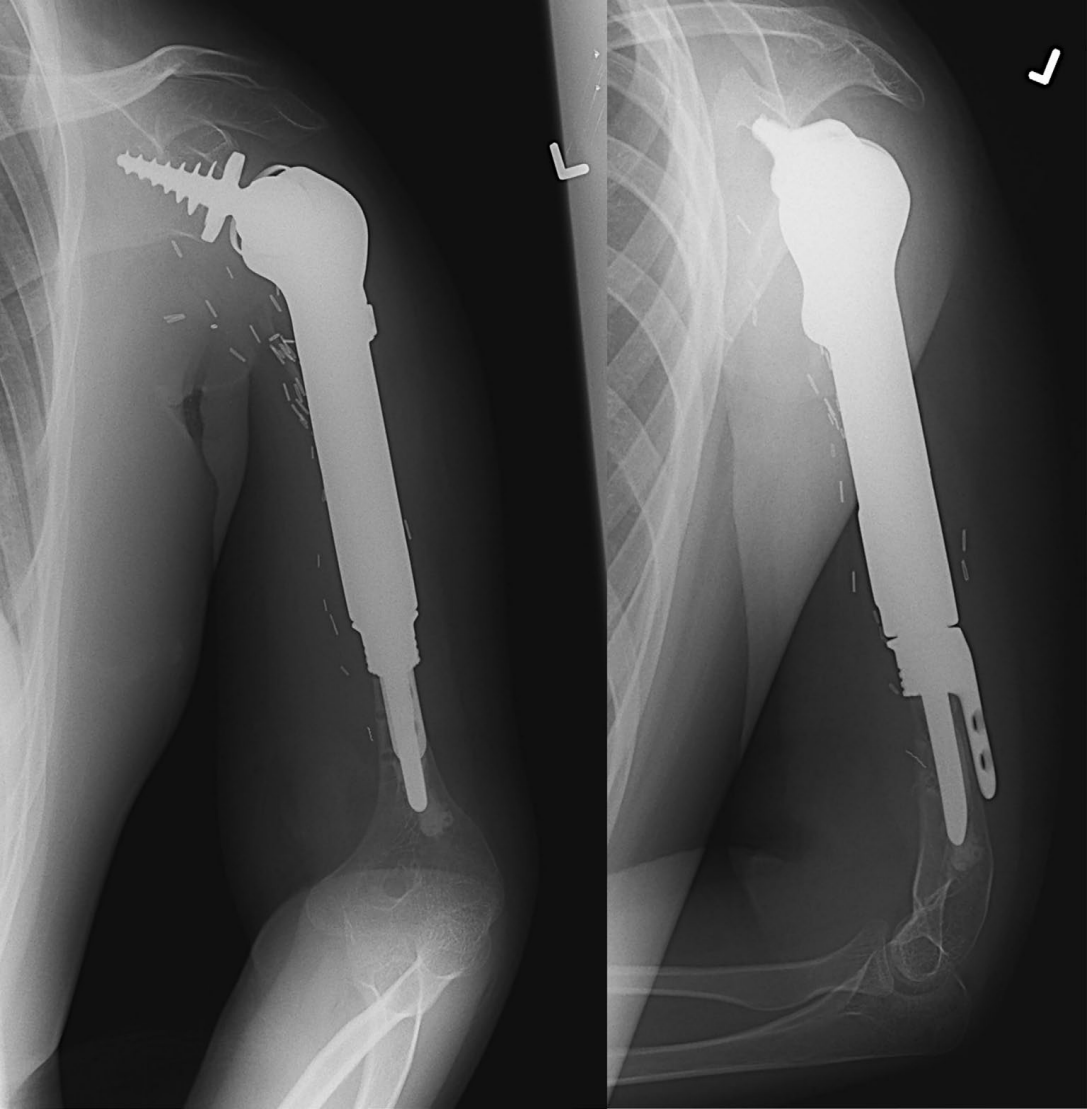
Immediate postoperative plain radiographs of patient in Figure 7 with a proximal humerus replacement with a growing (extendable) endoprosthesis and a HA‐coated stem collar with an extracortical plate.

**FIGURE 9:**
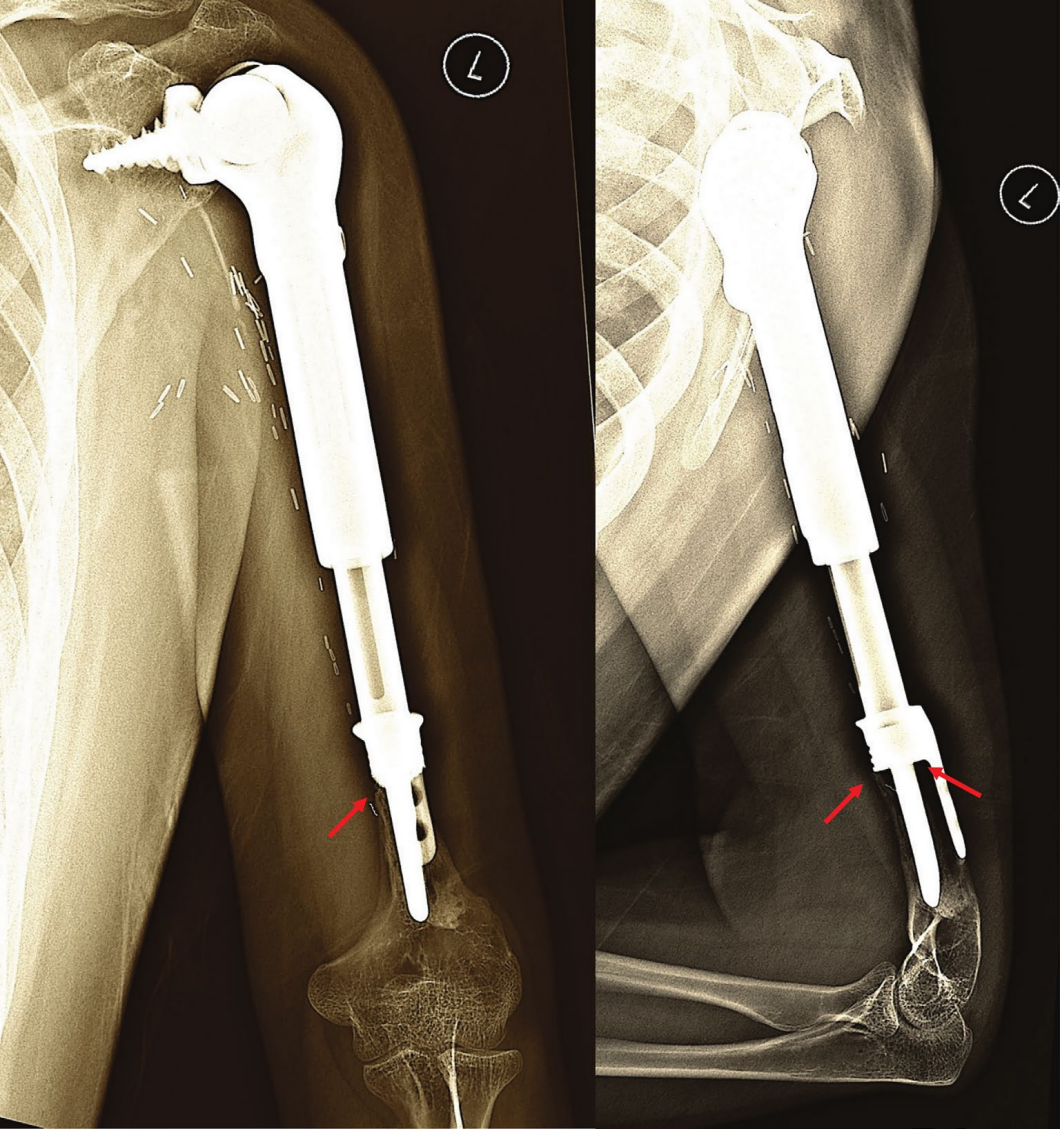
AP and lateral views of the most recent plain radiographs of patient in Figure 7 with the growing (extendable) endoprosthesis showing contact between the cortical bone and stem collar (red arrows) but no extracortical bone bridge ingrowth. However, no radiolucent lines are present to suggest loosening.

The primary outcomes of interest were assessing radiographic osseointegration at the bone‐implant interface and the rate of aseptic loosening. Secondary endpoints were complications along with clinical, functional and oncologic outcomes. Aseptic loosening and osseointegration were evaluated clinically and radiographically. Pain around the bone‐implant junction and start‐up pain were clinical indicators. Meanwhile, radiographic outcomes were evaluated by assessing stem migration, bone‐to‐implant alignment changes and bone ingrowth. Extracortical bone bridge ingrowth into the HA‐coated stem collar was documented on both AP and lateral plain radiographic views. Four zones were defined: medial and lateral on the AP view, anterior and posterior on the lateral view. For each zone, a score of one was given when bone ingrowth into the collar grooves was present. Of note, if there was contact between the collar and cortical bone but no evidence of additional growth into the collar groove(s), a score of zero was given; see Figures 5, 6, 7, 8, 9. The maximum score was four. A radiolucent line between the implant and bone received a score of zero [5]. All radiographs were reviewed by both authors, where stem migration and bone‐to‐implant alignment were determined by comparing the first and most recent postoperative plain radiographs. Clinical outcomes were evaluated based on qualitative pain (none, mild, moderate, severe), range of motion, limb‐length discrepancy (LLD) and gait aid at the final follow‐up. Functional outcomes were assessed using the Musculoskeletal Tumor Society (MSTS) scoring system. Oncologic outcomes, including local recurrence, metastatic disease and overall survival, were also reported.

### 2.3 Statistical Analysis

Statistical analysis was performed using GraphPad Prism 10 (San Diego, California, USA). Continuous and categorical variables were presented as mean (with range) and frequencies (with percentages), respectively. The Student's t‐test (or Mann–Whitney test in non‐parametric data) for continuous variables and the Fischer's Exact test for categorical variables were used to evaluate associations between extracortical bone bridge ingrowth scores and baseline characteristics as well as postoperative complications. Pre‐ and postoperative clinical outcomes were also compared. Statistical significance was reached when a two‐tailed *p*‐value was less than 0.05.

## 3 Results

Initially, 18 patients met the inclusion criteria. However, three patients were excluded as they resided in foreign countries and were followed by their local orthopaedic surgeon upon discharge from surgery. Of the 15 patients included, seven were women, and eight were men. The mean age at the time of final revision was 23 years (range six to 44 years). Six patients had multiple revisions. The mean time between the index procedure and final revision was 96 months (range 24 to 217 months), and the mean follow‐up time was 12 years (range 6.5 to 18 years). Index tumour resection locations include the distal femur (*n* = 10), proximal tibia (*n* = 2), midshaft femur (intercalary) (*n* = 1) and proximal humerus (*n* = 2). The extracortical plates in the lower extremity revisions measured 50 mm in nine of 13 patients. Plate length variations of 36 mm, 40 mm and 55 mm (*n* = 2) were designed as a result of anatomic and/or screw placement considerations during custom endoprosthesis planning. Upper extremity extracortical plate lengths were 25 mm and 30 mm. See Table 1 for baseline characteristics, oncologic history, surgical history and final surgical/implant information.

**TABLE 1:** Baseline characteristics, oncologic history, surgical history and final implant information.

Mean age at index surgery	14 years (range 3 to 42)
**Sex**	
Female	7 (47%)
Male	8 (53%)
**Location**	
Distal femur	10 (67%)
Proximal tibia	2 (13%)
Mid femur (intercalary)	1 (7%)
Proximal humerus	2 (13%)
**Index diagnosis**	
Osteosarcoma	12 (80%)
Ewing sarcoma	1 (7%)
Giant cell tumour of bone	2 (13%)
**Mean index tumour length**	17.7 cm (range 9 to 23)
**Mean index tumour volume (*n* = 8)**	391.8 mL (range 42 to 921)
**Index procedure type**	
Endoprosthesis	2 (13%)
Extendable endoprosthesis	2 (13%)
Allograft reconstruction	9 (60%)
Fibula VBG	1 (7%)
Curettage	1 (7%)
**Previous chemotherapy**	
Yes	13 (87%)
No	2 (13%)
**Previous radiation**	
Yes	0
No	15 (100%)
**Mean age at final revision**	23 years (range 6 to 44)
**Mean time between index procedure and final revision**	96 months (range 24 to 217)
**Multiple revisions before the final revision**	
Yes	6 (40%)
No	9 (60%)
**Reason for final revision**	
Allograft non‐union	4 (27%)
VBG fracture	2 (13%)
Endoprosthetic aseptic loosening	3 (20%)
Endoprosthetic joint instability/dislocation	2 (13%)
Endoprosthetic joint infection	1 (7%)
Endoprosthetic stem fracture	1 (7%)
Severe disability	2 (13%)
**Final revision implant type**	
Non‐extendable endoprosthesis	9 (60%)
Extendable endoprosthesis	5 (33%)
Intercalary endoprosthesis	1 (7%)
**Extracortical plate method of fixation**	
None	9 (60%)
Cable	5 (33%)
Screw	1 (7%)
**Mean stem‐to‐bone ratio**	0.51 (range 0.35 to 0.69)
**Stem length/bone segment**	
Short‐segment (< 100 mm)	9 (60%)
Standard length	6 (40%)
**Mean planned additional osseous resection**	29.4 mm (range 0 to 113)
**Mean residual native bone length**	122 mm (range 65 to 220)
**Mean extracortical plate length‐to‐residual native bone length ratio**	0.42 (range 0.23 to 0.63)
**Junctional bone graft**	
Cortical and cancellous autograft	5 (33%)
Cancellous autograft alone	8 (53%)
None	2 (13%)

Abbreviations: cm: centimetres, mL: millilitres, mm: millimetres, VBG: vascularized bone graft.

### 3.1 Osseointegration and Aseptic Loosening

At the final follow‐up, all patients achieved radiographic osseointegration, and none had clinical or radiographic evidence of aseptic loosening. No patient had pain at the bone‐implant junction or start‐up pain. Radiographically, no stem migration, no changes in bone‐to‐implant alignment and no radiolucent lines were identified in the final plain radiographs. Extracortical bone bridge ingrowth scores were 4/4 (*n* = 3), 3/4 (*n* = 4), 2/4 (*n* = 3) and 1/4 (*n* = 3) while two patients had a score of zero. All but two patients had at least cortical bone contact with the collar in all four zones, whereas the other two had contact in three out of four zones. The distal femur location (*p* = 0.044), endoprosthesis (as opposed to biologic reconstruction) as the index reconstruction method (*p* = 0.026), endoprosthetic mechanical failure (as opposed to endoprosthetic non‐mechanical failure and biologic reconstruction failure) as the reason for final revision (*p* = 0.0047) and additional fixation to the extracortical plate (*p* = 0.041) were associated with higher extracortical bone bridge ingrowth scores, see Table 2.

**TABLE 2:** Associations between extracortical bone bridge scores and baseline characteristics as well as postoperative complications.

Variable	EBB Score 0–2 (*n* = 8)	EBB Score 3–4 (*n* = 7)	*p*‐value
**Mean age at index surgery**	11 years (range 3 to 21)	18 years (9 to 42)	0.19
**Sex**			
Female	3 (42.9%)	4 (57.1%)	0.62
Male	5 (62.5%)	3 (37.5%)	
**Location**			
Distal femur	3 (30%)	7 (70%)	0.044*
Proximal tibia	2 (100%)	0	
Mid femur (intercalary)	1 (100%)	0	
Proximal humerus	2 (100%)	0	
**Index diagnosis**			
Osteosarcoma	6 (50%)	6 (50%)	> 0.99
Ewing sarcoma	1 (100%)	0	
Giant cell tumour of bone	1 (50%)	1 (50%)	
**Mean index tumour length**	17.6 cm (range 9–23)	17.9 cm (range 13–22)	0.92
**Mean index tumour volume (*n* = 8)**	429.0 mL (range 42 to 921)	280.0 mL (range 230–330)	0.58
**Index procedure type**			
Endoprosthesis	0	4 (100%)	0.026*
Biologic Reconstruction	8 (73%)	3 (27%)	
**Previous Chemotherapy**			
Yes	6 (50%)	6 (50%)	> 0.99
No	2 (67%)	1 (33%)	
**Mean age at final revision**	19 years (range 6 to 27)	27 years (11 to 44)	0.16
**Mean time between index procedure and final revision**	89 months (range 25–199)	103 months (24 to 217)	0.68
**Multiple revisions before the final revision**			
Yes	3 (50%)	3 (50%)	> 0.99
No	5 (56%)	4 (44%)	
**Reason for final revision**			
Endoprosthetic mechanical failure [22]	0	6 (100%)	0.0047*
Endoprosthetic non‐mechanical failure [22]	1 (100%)	0	
Biologic reconstruction non‐union or fracture	5 (83%)	1 (17%)	
Severe disability	2 (100%)	0	
**Final revision implant type**			
Non‐extendable endoprosthesis	5 (50%)	5 (50%)	> 0.99
Extendable endoprosthesis	3 (60%)	2 (13%)	
**Extracortical plate method of fixation**			
None	7 (78%)	2 (22%)	0.041*
Cable or screw	1 (17%)	5 (83%)	
**Mean stem‐to‐bone ratio**	0.49 (range 0.44 to 0.62)	0.53 (range 0.35 to 0.69)	0.45
**Mean stem length**	87.3 mm (38 to 130)	93.4 mm (range 54 to 140)	0.72
**Mean stem diameter**	10.9 mm (range 7 to 19)	11.8 mm (range 10 to 15)	0.57
**Stem length/bone segment**			
Short‐segment (< 100 mm)	5 (56%)	4 (44%)	> 0.99
Standard length	3 (50%)	3 (50%)	
**Mean residual native bone length**	119 mm (65 to 220)	125 mm (80 to 193)	0.80
**Mean extracortical plate length‐to‐residual native bone length ratio**	0.40 (range 0.23 to 0.56)	0.44 (0.26 to 0.63)	0.61
**Junctional bone graft**			
Cortical and cancellous autograft	1 (20%)	4 (80%)	0.12
Cancellous only or none	7 (70%)	3 (30%)	
**Complications**			
Yes	4 (80%)	1 (20%)	0.28
No	4 (40%)	6 (60%)	

*Note:* They highlight values that are statistically significant.

Abbreviations: cm: centimetres, EBB: extracortical bone bridge, mL: millilitres, mm: millimetres, (%) represents row percentages, *: statistically significant.

### 3.2 Complications, Clinical, Functional and Oncologic Outcomes

Five of the included patients sustained postoperative complications. One patient had an extendable distal femur replacement, where the telescoping mechanism failed seven years later and was revised with a rigid component. One patient with an epiphyseal‐sparing proximal humerus replacement was affected due to epiphyseal bone resorption and implant dissociation proximally, which was revised to a total shoulder replacement six years later. Acute prosthetic joint infection (PJI) was found in two patients, including one proximal tibia replacement and one distal femur endoprosthesis, where the latter patient also suffered a gastrocnemius flap necrosis. Both underwent irrigation and debridement (I&D) with modular components exchange, and the latter patient also underwent soft tissue flap revision with no further complications. None of these patients had their HA‐coated stem collar with the extracortical plate removed due to their complications, and only portions of the custom implants were revised. No evidence of aseptic loosening was observed at the final follow‐up. One patient with a complex medical history had a chronic PJI following revision to an extendable distal femur replacement. Along with antibiotic suppression, multiple I&Ds were performed, but the patient refused any staged revision. As the infection evolved, there was evidence of chronic tibia osteomyelitis, and amputation also became an option after multidisciplinary team discussion. The patient wished to proceed with an above‐knee amputation. The HA‐coated stem collar with extracortical plate was not removed to maximize the stump size; there was no evidence of infection at that level. Intraoperatively, the stem was noted to be well‐fixed.

Clinically, 13 patients were pain‐free at the final follow‐up, and two patients had mild pain with activities but did not involve the region around the bone‐implant junction. All patients could perform their normal activities of daily living, 13 were back to their normal work, and three were playing high‐intensity or highly competitive sports. Gait aids were used by two patients for long‐distance ambulation only. Excluding the amputated patient, the mean joint range of motion and LLD significantly improved for patients undergoing lower extremity revisions (*p* < 0.0001 and *p* = 0.021, respectively); see Table 3. The mean MSTS score for all patients was 26.1 (range 19 to 30). Everyone, except for the patient who eventually had an amputation, scored 23 or greater (graded excellent) [21].

**TABLE 3:** Comparison of pre‐ and postoperative clinical outcomes.

	Preoperative	Postoperative	*p*‐value
Lower Extremity (*n* = 12)^a^			
Mean knee ROM (arc of motion)	45.8 deg. (range 10 to 100)	104.2 deg. (range 75 to 130)	< 0.0001*
Mean limb‐length discrepancy	3.4 cm (range 0 to 6)	1.3 cm (range 0 to 6)	0.021*
**Upper Extremity (*n* = 2)**			
Mean shoulder FF (arc of motion)	35.0 deg. (range 10 to 60)	40.0 deg. (both 40)	0.86
Mean limb‐length discrepancy	5.0 cm (range 0 to 10)	2.5 cm (range 0 to 5)	0.70

*Note:* They highlight values that are statistically significant.

Abbreviations: ROM: range of motion, FF: forward flexion, deg.: degrees, cm: centimetres, *: statistically significant.

^a^ Excludes the patient who eventually underwent above‐knee amputation.

All patients remain alive with no evidence of active disease. Twelve patients were disease‐free following the index procedure. One patient had a local recurrence of a giant cell tumour of the proximal tibia, which was revised to a proximal tibia replacement with no evidence of recurrence. Two patients who were treated for osteosarcoma presented with a solitary lung metastatic lesion. Both underwent lung wedge resections and additional chemotherapy with no evidence of disease at the most recent surveillance imaging.

## 4 Discussion

Aseptic loosening following tumour reconstruction is a significant issue, especially in young patients, due to levels of physical activity, inadequate cementation, stem length and diameter, poor fixation in short‐segment stems and constrained implants, thereby increasing bone‐implant stress [7, 10, 11, 22, 23, 24]. The findings of this study demonstrate that utilizing a HA‐coated stem collar with an extracortical plate provides a reliable long‐term solution against aseptic loosening at the bone‐implant interface, especially for revision (and multiple revision) endoprosthetic reconstructions as well as for revision short‐segment stems. None of the patients had evidence of aseptic loosening. Even among those with various non‐type II complications as per the types of endoprosthetic failure described by Henderson et al. (2011) [22], none of the patients required stem revision.

The results correlated with previously published data. Stevenson et al. (2017) reported 37 patients with a custom short‐segment HA‐coated stem collar and extracortical plate, of which 12 were primary surgeries and 25 were revisions. Only three of eight complications were related to aseptic loosening, occurring at a mean of 7.7 years [7]. Tsuda et al. (2019) reviewed 18 paediatric patients who underwent physis‐preserving endoprosthetic reconstruction where ≤ 5 cm of bone stock remained between the resection level and the physis. These patients had custom HA‐coated collars with extracortical plate +/− short stems. Only one patient had aseptic loosening as a complication at a median follow‐up of 67‐month follow‐up [25]. A study of 14 patients with short‐segment proximal femora, who underwent endoprosthetic reconstruction with stem side‐plate and interlocking screws into the native femoral head and neck, only had two failures due to aseptic loosening at a nearly mean follow‐up of 5‐year follow‐up [26]. Thus, with the longest follow‐up period for this implant specification, this study has demonstrated that this construct provides a valid and reliable long‐term solution against aseptic loosening in the setting of revisions and revision for short‐segment bone stock; see Table 4.

**TABLE 4:** Comparison between previous publications and the current study assessing extracortical plate fixation in **short‐segment** endoprosthetic reconstruction.

Authors & Year	Number of patients	Age	Location(s) of reconstruction	Mean stem length	Follow‐up	* Failure type [22]	Short‐segment survivorship
Christ et al. 2021 [26]	14	Mean 36 +/− 14 years	DF: 10, Intercalary femur: 4; all proximal femora short segment (4 primaries, 10 revisions)	24% or less of the femur length	Mean 4.7 +/− 3.3 years	Type I: 1 Type II: 2 Type III: 2 Type IV: 1	Two failures of the short‐segment fixation requiring revision
Stevenson et al. 2017 [7]	37	Mean 28.5 years (range 7 to 86)	PH: 9, PF: 9, DF: 13, PT: 6 (12 primary, 25 revisions)	Humerus: 33 mm (range 20 to 60); Lower limb: 79 mm (range 34 to 100)	Mean 7 years (range 1 to 17)	Type II: 3 Type III: 2 Type IV: 3	No aseptic loosening with extracortical plate osseointegration (*n* = 27); Without plate osseointegration (*n* = 10), three aseptic loosening
Tsuda et al. 2019 [25]	18	Median 10 years	Femur: 11, PH: 6, PT: 1 (17 primary, 1 revision)	5 cm or less, physis‐preserving metadiaphyseal stems	Median 67 months	Type II: 1 Type III: 2	One aseptic loosening
Cobb et al. 2005 [24]	3	—	MH: 1, DH: 1, DF: 1 (3 primaries)	Stemless triplate fixation	Mean 26 months (range 11 to 53)	—	2 patients died of pulmonary metastasis
Current Study	9	Mean 25 years (range 6 to 44)	PH: 2, Intercalary femur: 1, DF: 5, PT: 1 (all revisions)	PH: 39 mm (range 38–40) Lower limb: 78 mm (range 54 to 90)	Mean 14 years (range 8 to 18)	Type III: 1 Type IV: 1	No aseptic loosening

Abbreviations: DF: distal femur, DH: distal humerus, MH: midshaft humerus, PF: proximal femur, PH: proximal humerus, PT: proximal tibia.

* Failure type: according to the classification of endoprosthetic modes of failures described by Henderson et al. (2011) [22].

Limb salvage surgery in musculoskeletal oncology, when feasible, is preferred over amputations due to its favourable functional outcomes and lack of survival benefits with amputations. However, the complication rate remains high, where subsequent revisions are a major risk factor for further complications and decreased functional scores [27, 28, 29]. Given the high burden of aseptic loosening in tumour endoprosthetic reconstructions, which continue to increase in patients with long‐term survival and/or re‐revisions, the objective of achieving greater extracortical bone bridge ingrowth scores is to improve long‐term stem fixation survivorship. Extracortical bone bridging allows for reduced bone‐implant stresses and improved stress distribution. It has also been shown to improve torsional stiffness while preventing stress shielding and osteolysis [4, 18, 30, 31].

Although previous studies have suggested that a low extracortical bone bridge ingrowth score or a score of two or less increases the risk of aseptic loosening [4, 5], this study, with a nearly equal distribution of patients with scores 0 to 2 and 3 to 4, had no reported aseptic loosening. It is hypothesized that this is due to the stability provided by the extracortical plate at the bone‐implant junction by decreasing stem micromotion, which is a major factor in causing aseptic loosening [7, 16, 32]. This hypothesis is supported by the fact that where there was no extracortical bone ingrowth into the HA‐coated stem collar grooves, there was at least contact between the cortical bone and stem collar at the bone‐implant interface without radiographic evidence of radiolucent lines or clinical symptoms related to aseptic loosening at long‐term follow‐up [4, 5, 8]. Furthermore, in this study, the humeri reconstructions (*n* = 2) had extracortical bone bridge ingrowth scores of zero and one. Upper extremity aseptic loosening is less common than in the lower extremity, possibly due to the differences in load stressing related to weight bearing. Therefore, outcomes may not be as affected by extracortical bone bridge ingrowth as in the lower extremity [33]. Despite these results, the objective of adding HA coating and an extracortical plate is to obtain immediate stability and to promote bone ingrowth and extracortical osseous apposition, thereby decreasing the risk of long‐term aseptic loosening [5]. However, larger and prospective studies are necessary to determine the true predictive effects of each extracortical bone bridge ingrowth score on aseptic loosening prevention.

In the univariate analysis of this study, distal femur surgeries, additional fixation to the extracortical plate (screws or cables), index endoprosthetic reconstruction procedures as opposed to index biologic reconstructions, and mechanical failures as opposed to non‐mechanical and biologic failures were associated with statistically significantly higher extracortical bone bridge ingrowth scores. Murugan et al. (2021) demonstrated superior extracortical bone bridge ingrowth score in distal femur reconstructions compared to the proximal tibia at two years (*n* = 29), although not statistically significant [18]. These differences may be accounted for by the less constrained hinge mechanism of the distal femur replacement compared to the proximal tibia, shorter segment fixation into the tibia and soft tissue coverage [10, 18]. Regarding the additional fixation to the extracortical plate, screw or cable fixation provides improved initial stability and osseomechanical integration. In addition to HA‐coated surfaces, this allows for enhanced bone formation, osseointegration and load sharing between the plate and bone, thus providing excellent short‐ and long‐term stability [16, 24, 34].

Patients who were revised to the custom endoprosthesis following endoprosthetic reconstruction failure, especially mechanical failure, had significantly better extracortical bone bridge scores. This is likely due to the nature of the failure. Most patients with a biologic reconstruction failure were revised due to non‐union with pseudarthrosis or graft fractures. Consequently, this often indicates an impaired biologic healing environment, which includes compromised vascularity, immune responses to the bone graft and soft tissue deficiency. This can be compounded by previous chemotherapy, radiation and infection. Meanwhile, endoprosthetic revisions were more commonly due to mechanical failures where poor biology likely contributed to a lesser degree. Therefore, it is hypothesized that patients with previous endoprosthetic reconstructions had better bone healing environments around the bone‐implant interface allowing for more extracortical bone formation as observed in this study [22, 35, 36, 37, 38]. Given that none of the patients demonstrated evidence of radiographic or clinical signs of aseptic loosening, this indicates that custom HA‐coated stem collar with an extracortical plate fixation is an excellent alternative solution to implant loosening, especially in patients with multiple significant risk factors.

The findings in this study of custom‐manufactured implants also demonstrate excellent clinical and functional outcomes. Only two patients had residual mild pain and used gait aids for long‐distance ambulation, while all others were pain‐free. The mean MSTS score was 26.1 (range 19 to 30). Other than the one patient who eventually underwent amputation, all patients had an excellent score, including the other four patients who had postoperative complications (two mechanical failures and two acute PJIs) [21]. Functional outcomes were similar to the current literature regarding tumour endoprosthetic reconstructions around the shoulder and knee joints, including primary surgeries and revisions [39, 40]. Furthermore, when feasible and long‐term survival is expected, the argument for joint‐sparing surgery is favoured in both revision and/or short‐segment cases, as functional outcomes tend to be higher [41].

The results showed a significant improvement in range of motion and LLD. While the range of motion is underreported, it was found to be comparable to several studies, including non‐expandable distal femur replacements (105°, *n* = 108) [42], skeletally mature cemented endoprosthesis around the knee (96.6°, *n* = 108) [43], and distal femur endoprosthetic revision to a Compress system (90°, *n* = 17) [12]. Although five patients had non‐Type II complications, the rate falls within the range of the current body of evidence for primary and revision surgeries [1, 14, 39]. Thus, the results demonstrate that custom solution implants provide excellent long‐term clinical and functional outcomes, especially in multiple revisions and short‐segment fixation settings, and can also be utilized to reduce LLD [44]. Furthermore, with the complications encountered, they also demonstrated that custom implants make partial exchange of modular components possible. Although custom implants are expensive and time‐consuming to design and manufacture, the investment has a role, especially if the financial cost outweighs the resource burden of further revision(s) from standard implants. Although this applies to a select patient base, further cost analysis is warranted [45].

This study's limitations stem from the disease's rarity, the selectiveness of patients undergoing this type of surgery, the retrospective design and reliance on medical records. Consequently, potential confounding factors may not be accounted for, and outcome differences may not be discriminated. In this study, all patients were disease‐free at the time of surgery and had an expected long‐term survival. Given the low numbers of midshaft femur (intercalary), proximal tibia and proximal humerus reconstruction, the data was combined with the distal femur endoprosthesis outcomes to increase power. In this regard, short‐segment revisions were also combined with standard stem lengths, although short‐segment fixation was non‐inferior to the latter. Additionally, there were no histologic analyses or computed tomography scans to further assess osseointegration, as previous studies have demonstrated possible discordance between radiographs and histologic analysis [5, 15]. Finally, the low sample size from a single‐institution study along with patient heterogeneity (including age, tumour type, size and grade, anatomic location, implant specification, as well as differences in the number and reasons for revisions) may affect the generalizability of the results within the broader and general population.

## 5 Conclusions

In this study, a custom HA‐coated stem collar with an extracortical plate fixation has demonstrated excellent long‐term results for enhancing bone‐implant osseointegration and reducing the rate of aseptic loosening in revision endoprosthetic reconstructions following initial bone tumour resections and reconstructions. There is a particular utility for short‐segment fixation where the native joint is preserved and for revision/multiple revisions, especially where bone quality is impaired (previous infection, decreased vascularity, soft tissue deficiency) and where risks of aseptic loosening are high. Furthermore, the extracortical plate allows for additional initial construct and rotational stability, potentially enhancing the bone‐implant osseointegration. However, given the rarity of the disease, the small sample size, the specific population subset and the heterogeneity of this study, larger scale and preferably multicentric studies are necessary to further validate the results.

## Author Contributions

**Patrick Qi Wang:** conceptualization (equal), data curation (lead), formal analysis (lead), methodology (equal), visualization (lead), writing – original draft (lead), writing – review and editing (lead). **Kwok Chuen Wong:** conceptualization (equal), investigation (lead), methodology (equal), supervision (lead), visualization (supporting), writing – original draft (supporting), writing – review and editing (supporting).

## Conflicts of Interest

The authors declare no conflicts of interest.

## Data Availability

The data that support the findings of this study are available from the corresponding author upon reasonable request.
